# Genetic basis of maize kernel starch content revealed by high-density single nucleotide polymorphism markers in a recombinant inbred line population

**DOI:** 10.1186/s12870-015-0675-2

**Published:** 2015-12-12

**Authors:** Tingting Wang, Min Wang, Shuting Hu, Yingni Xiao, Hao Tong, Qingchun Pan, Jiquan Xue, Jianbing Yan, Jiansheng Li, Xiaohong Yang

**Affiliations:** National Maize Improvement Center of China, Beijing Key Laboratory of Crop Genomics and Genetic Improvement, China Agricultural University, 100193 Beijing, China; College of Agronomy, Northwest A&F University, Yangling, Shaanxi 712100 China; National Key Laboratory of Crop Improvement, Huazhong Agricultural University, Wuhan, 430070 Hubei China

**Keywords:** Maize, Starch content, QTL, SNP, Bin map

## Abstract

**Background:**

Starch from maize kernels has diverse applications in human and animal diets and in industry and manufacturing. To meet the demands of these applications, starch quantity and quality need improvement, which requires a clear understanding of the functional mechanisms involved in starch biosynthesis and accumulation. In this study, a recombinant inbred line (RIL) population was developed from a cross between inbred lines CI7 and K22. The RIL population, along with both parents, was grown in three environments, and then genotyped using the MaizeSNP50 BeadChip and phenotyped to dissect the genetic architecture of starch content in maize kernels.

**Results:**

Based on the genetic linkage map constructed using 2,386 bins as markers, six quantitative trait loci (QTLs) for starch content in maize kernels were detected in the CI7/K22 RIL population. Each QTL accounted for 4.7 % (*qSTA9-1*) to 10.6 % (*qSTA4-1*) of the starch variation. The QTL interval was further reduced using the bin-map method, with the physical distance of a single bin at the QTL peak ranging from 81.7 kb to 2.2 Mb. Based on the functional annotations and prior knowledge of the genes in the top bin, seven genes were considered as potential candidate genes for the identified QTLs. Three of the genes encode enzymes in non-starch metabolism but may indirectly affect starch biosynthesis, and four genes may act as regulators of starch biosynthesis.

**Conclusions:**

A few large-effect QTLs, together with a certain number of minor-effect QTLs, mainly contribute to the genetic architecture of kernel starch content in our maize biparental linkage population. All of the identified QTLs, especially the large-effect QTL, *qSTA4-1*, with a small QTL interval, will be useful for improving the maize kernel starch content through molecular breeding.

**Electronic supplementary material:**

The online version of this article (doi:10.1186/s12870-015-0675-2) contains supplementary material, which is available to authorized users.

## Background

Maize is a leading crop worldwide because of its diverse functions as a source for human food and animal feed and as a raw material for industry and manufacturing. With a growing world population and need for biofuel, increasing maize grain yield is necessary to meet the market demand. Starch is the major component of maize kernels, accounting for 70 % of the kernel weight. In addition, starch is increasingly used as a renewable chemical feedstock for the conversion of other products, such as high fructose corn syrup, polymer-based fibers and fuel ethanol [1]. Therefore, the ability to manipulate starch quality and quantity in maize kernels is an important goal in maize breeding.

Starch is deposited as water-insoluble semicrystalline granules, which are chemically comprised of two homopolymers of α-d-glucose, amylose and amylopectin, in the maize endosperm. Although starch metabolism is complex, it is clear that four classes of enzymes, adenosine diphosphate glucose pyrophosphorylases (AGPases), starch synthases (SSs), starch branching enzymes (SBEs) and debranching enzymes (DBEs), play critical roles in starch biosynthesis. Maize mutants have been used to isolate genes encoding key enzymes in starch metabolism, such as *Shrunken1* (*sh1*), *Shrunken2* (*sh2*), *Brittle2* (*bt2*), *agpsemzm*, *agpllzm*, *Waxy1* (*wx1*), *SS1*, *Sugary2* (*su2*), *Dull1* (*du1*), *SS2b-2*, *SS2c*, *SS3b-1*, *SS3b-2*, *SS4*, *SBEIa*, *SBEIIa*, *Amylose extender1* (*ae1*) and *Sugary1* (*su1*) [2, 3]. The *sh1* gene encodes the major isoform of sucrose synthase and provides an important link in sucrose-starch conversion reactions, as sucrose synthase catalyzes the reversible reaction between sucrose and uridine diphosphate-glucose [4]. *sh2*, *bt2*, *agpsemzm* and *agpllzm* encode the large or small subunits of AGPase, which converts glucose-1-phosphate to ADP-glucose, the precursor for starch synthesis [3, 5–7]. *wx1*, encoding granule-bound SS I, is solely responsible for amylose production, whereas *SS1*, *Su2*, *Du1, SS2b-2*, *SS2c*, *SS3b-1*, *SS3b-2* and *SS4*, encoding four types of soluble SS, are responsible for amylopectin production [8–13]. *SBEIa*, *SBEIIa* and *ae1* encode the SBE isoforms Ia*,* IIa and IIb, respectively [14–16], which are all responsible for amylopectin production. *su1* encodes a DBE of the isoamylase type, and mutant *su1* kernels contain the highly branched, water-soluble phytoglycogen and constitute the original sweet corns [17]. These are the key steps in maize starch metabolism, but how they are connected still requires clarification. In addition, little is known regarding the regulation of starch biosynthesis and accumulation in maize.

QTL mapping is a classical method for identifying loci for quantitative traits of interest without prior genetic knowledge. A variety of QTLs for the starch content in maize kernels have been identified in different biparental populations since the first study in the Illinois High Protein × Illinois Low Protein F_3_ population, which was derived from a cross of two lines divergently selected for protein content after 76 generations in the Illinois long-term selection experiment [18–32]. Among these studies, 33 and 127 single nucleotide polymorphisms (SNPs) associated with starch content in maize kernels were further identified using the single regression method and the subsampling method in a nested association mapping population, respectively [28]. This information extended the limited knowledge regarding the causative genetic factors underlying QTLs of kernel starch content.

QTL mapping is firstly suggestive to identify loci for complex quantitative traits, although, the resolution is rather low, often ranging from 10 to 30 cM [33]. Increasing the marker density is one way to improve QTL mapping resolution [34]. With the development of genomics and genotyping technologies, SNP markers have been used to increase marker density because of their low time consumption, low cost and high throughput. They have been widely applied to construct genetic linkage maps and in the QTL mapping of wheat, rice, sorghum and maize [35–38]. The growing marker density not only increases the number of co-segregating markers but also leads to the computational challenge of constructing an ultra-high-density linkage map. Therefore, constructing a “skeleton bin map”, which combines the co-segregating markers into one bin and separates adjacent bins based on single recombination events, is an effective approach for capturing all of the recombination events using saturated markers [39], which increases the power, accuracy and resolution needed to identify QTLs [40–46].

In this study, a maize CI7/K22 recombinant inbred line (RIL) population was developed and genotyped using the Illumina MaizeSNP50 BeadChip, which contains 56,110 SNPs. The kernel starch contents of this RIL and the parental lines were evaluated after being grown in three environments. The objectives were to (1) construct a high-density genetic linkage map using the inferred bins as markers, (2) dissect the genetic architecture of starch content in maize kernels of the CI7/K22 RIL population, (3) narrow down the position of the identified QTLs using the SNP bin map and (4) mine the candidate genes associated with starch content in the refined QTL interval.

## Results

### Phenotypic variation in kernel starch content

**Fig. 1:**
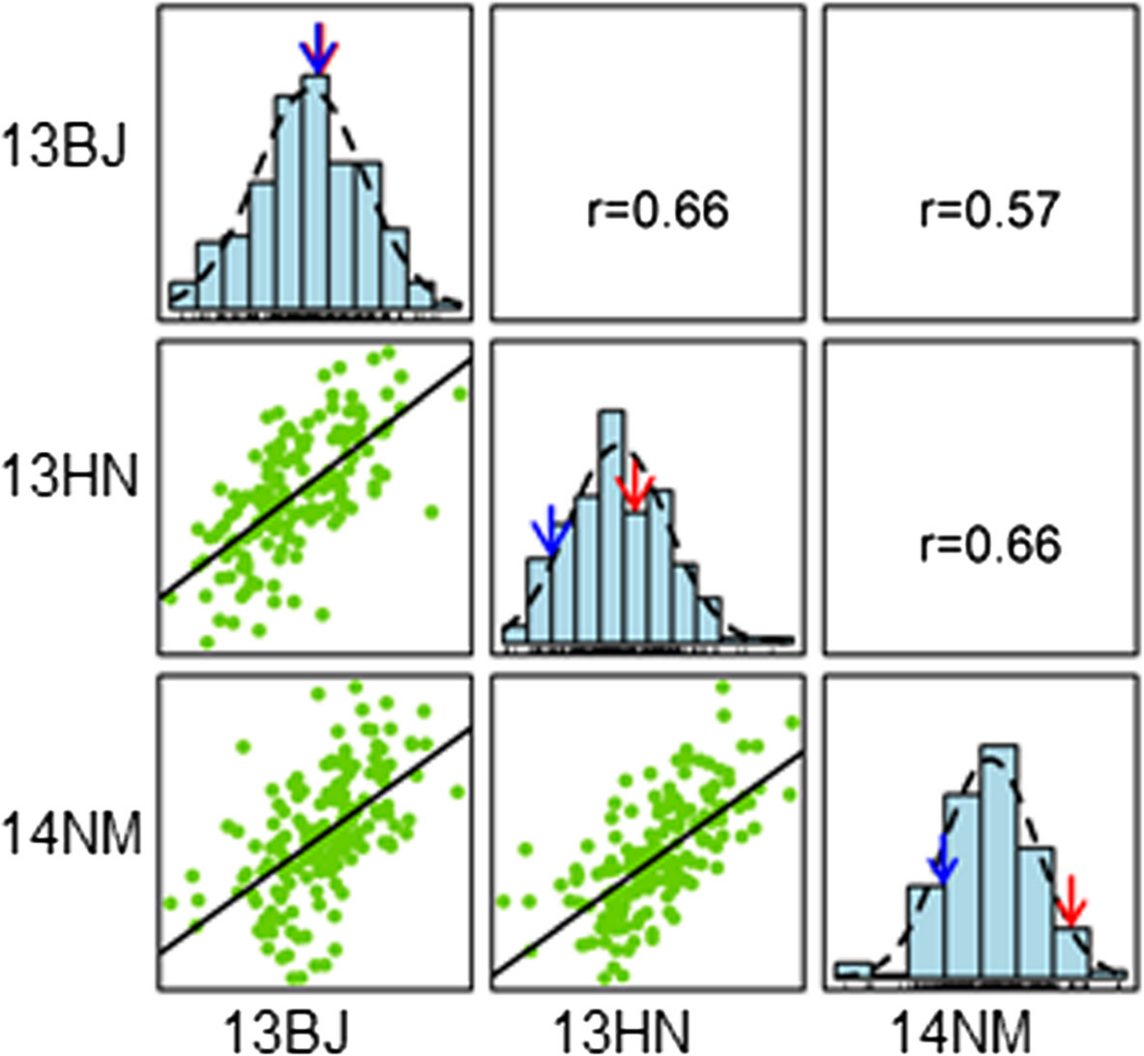
Frequency distribution of starch content in, and correlations across, three environments. The blue and red arrows represent the starch content of CI7 and K22, respectively. 13BJ, 13HN and 14NM represent the environments of Beijing in 2013, Hainan in 2013 and Neimeng in 2014, respectively

The low-starch inbred line CI7 has ~0.1 %, 3.5 % and 7.1 % lower starch content values than K22 in Beijing in 2013, Hainan in 2013 and Neimeng in 2014, respectively. Taken together, no significant difference was observed in the starch content between the two parents, CI7 and K22 (t = 2.13, *P* = 0.09). There were moderately positive starch content correlations among the three environments, with correlation coefficients ranging from 0.57 to 0.66 (Fig. 1). The Best Linear Unbiased Prediction (BLUP) value of the starch content revealed that the mean of the CI7/K22 RIL population was close to the mid-parent value (Table 1). A normal distribution was observed for the starch content with transgressive segregation in all environments (Fig. 1), indicating that the alleles responsible for increasing the starch content reside in both parents. The ANOVA results indicated that there were highly significant effects on the starch content that were due to genotype and environment (Table 1). The broad-sense heritability (*h*^*2*^) estimate of the starch content was high (82.1 %), indicating that much of the phenotypic starch content variation in the RIL population was genetically determined.

**Table 1:** Starch content data in the parental maize lines CI7 and K22 and the CI7/K22 RIL population

Items	CI7	K22	RIL
Mean ± SD (%)	63.16 ± 1.71	66.72 ± 1.62	64.82 ± 1.64
Range (%)	–	–	61.36–69.52
Variance: Genotype	–	–	2.95**
Variance: Environment	–	–	0.13**
Variance: Error	–	–	1.93
Heritability (%)(CI)^a^	–	–	82.1(78.0–85.5)

**, significant at *P* < 0.01

^a^Heritability, broad-sense heritability (*h* ^*2*^); CI, confidence

### Construction of bin and genetic linkage maps

**Fig. 2:**
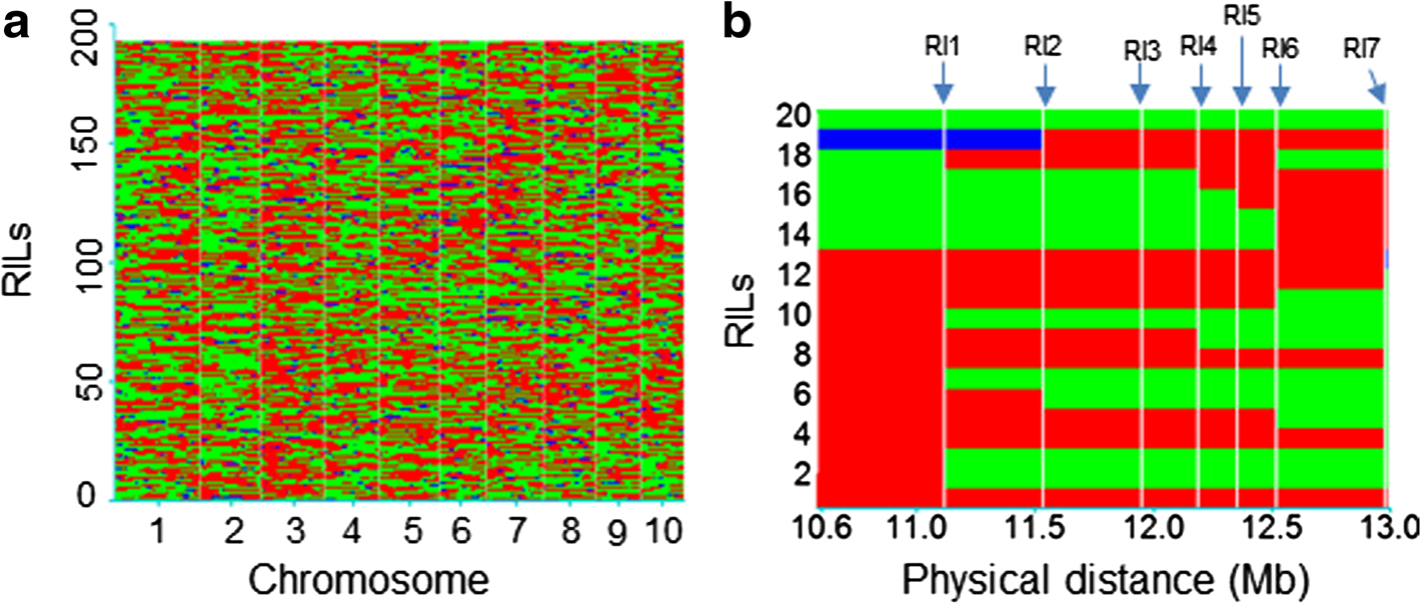
Recombination bin map of 210 RILs. **a** Genetic constituents of 210 lines in the K22/CI7 RIL population. Chromosomes are separated by vertical lines. Red, green and blue represent the K22, CI7 and heterozygous genotype, respectively. **b** An enlarged bin map showing part of chromosome 10, ranging from 10.6 Mb to 13.0 Mb, in 20 RILs. The white vertical lines represent recombination intervals (RIs), which are defined as the transition regions between two haplotype blocks in at least one of the 210 RILs. The chromosome fragment between two adjacent RIs was defined as a bin, which was used as a molecular marker

The CI7/K22 RIL population, which consists of 210 RILs, and both parental lines were genotyped using 56,110 SNPs. A total of 13,433 SNPs, with their precise physical positions based on the B73 reference sequence Version 5b.60 (http://ensembl.gramene.org/Zea_mays/Info/Index), were polymorphic between the two parents. The missing rate of these SNPs ranged from 0 to 15.31 %, with an average of 1.18 %, the heterozygosity ranged from 0 to 15.64 %, with an average of 3.69 % and the minor allele frequency ranged from 0.27 to 0.50, with an average of 0.45 in the CI7/K22 RIL population (Additional file 1). For all of the RILs, the missing rate in each line averaged 1.18, with a range of 0.09 to 28.93 %, and the heterozygosity in each line averaged 3.71, with a range of 0.04 to 19.41 % (Additional file 1). Based on these individual SNPs, bin maps were constructed for all 210 RILs, and the co-segregating markers in two contiguous block borders were lumped as a bin, resulting in a skeleton bin map consisting of 2,386 recombinant bins distributed throughout the genome (Fig. 2). The number of bins on each chromosome ranged from 148 to 392, and the physical lengths of the bins ranged from 0.34 kb to 44.2 Mb, with an average of 0.9 Mb (Additional file 1). In total, 79.4 % of the bins were less than 1 Mb in length, with 7.8 % of the bins being longer than 2 Mb (Additional file 1). Using each bin as a marker, the genetic linkage map of the CI7/K22 RIL population was constructed based on the recombination frequency. The total length of the linkage map for the CI7/K22 RIL population was 1,719.7 cM, with an average interval of 0.72 cM between adjacent bins (Additional file 1).

#### Identification of QTLs for starch content

**Fig. 3:**
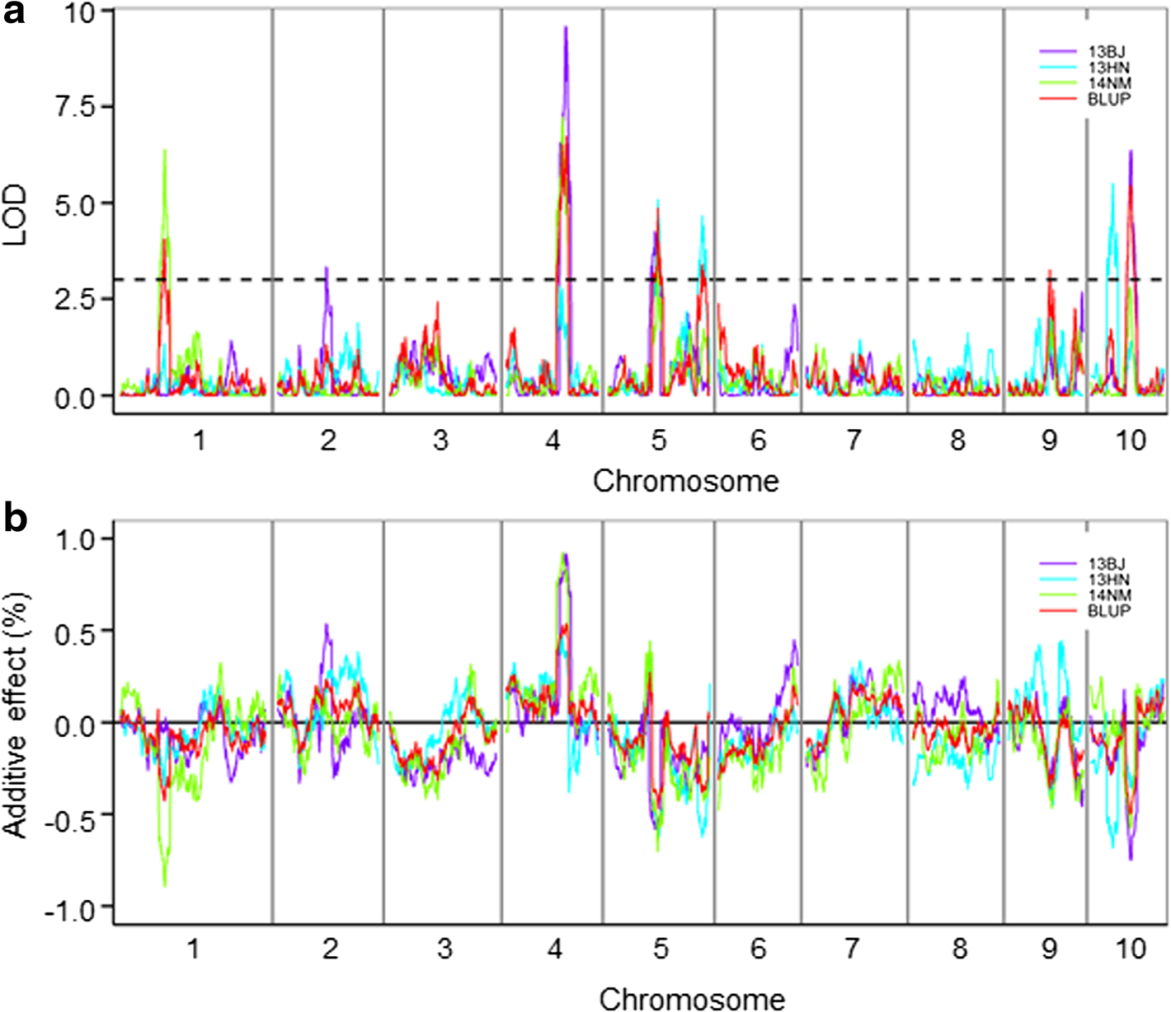
The distribution of starch content QTLs across the entire genome in different environments. **a** The LOD profiles of starch content QTLs. **b** The additive effects of starch content QTLs. 13BJ, 13HN and 14NM represent the environments of Beijing in 2013, Hainan in 2013 and Neimeng in 2014, respectively. BLUP represents the results of QTL mapping using the BLUP value of starch content based on three environments

Based on a linkage map of 1,719.7 cM, QTLs for starch content were first identified using the BLUP value across the three environments. In total, six QTLs controlling starch content were detected in the CI7/K22 RIL population at an empirical threshold logarithm of odds (LOD) value of 3.1 after 1,000 permutations (Table 2; Fig. 3). These QTLs were distributed among six genomic regions on chromosomes 1, 4, 5, 9 and 10. The QTL interval averaged 4.5 Mb (5.7 cM) with a range of 2.4 Mb to 8.7 Mb (2.1–13.1 cM). The starch variation in this RIL population that could be explained by all of the detected QTLs was 48.6 %, with each QTL ranging from 4.7 % (*qSTA9-1*) to 10.6 % (*qSTA4-1*). Alleles from K22, the high-starch parent, at all of the mapped loci except *qSTA4-1*, had increasing effects on the starch content. The largest QTL, *qSTA4-1*, was located on chromosome 4 and was flanked by PZE104103541 and PZE104106157. The CI7 allele at this locus had an additive effect of 0.54 % for increased starch content. The second largest QTL for starch content, *qSTA10-1*, located in the genomic region between bins SYN23550 and SYN22965, explained 9.1 % of the phenotypic variation, with an additive effect of 0.50 % on chromosome 10. The next two QTLs for starch content, *qSTA5-1* and *qSTA5-2*, were both located on chromosome 5 and explained 7.7 % and 5.3 % of the phenotypic variation, respectively.

**Table 2:** Individual starch content QTLs in the CI7/K22 RIL population

QTL	Chr	Marker interval	Genetic interval (cM)	Physical interval (Mb)^a^	LOD	Additive effect^b^	R^2^ (%)^c^
*qSTA1-1*	1	PZE101049395–PZE101053646	76.6–80.6	34.0–37.7	4.07	−0.42	6.2
*qSTA4-1*	4	PZE104103541–PZE104106157	104.8–109.2	179.8–182.3	6.75	0.54	10.6
*qSTA5-1*	5	PZE105100606–PZE105105086	87.9–90.0	150.8–159.5	4.87	−0.47	7.7
*qSTA5-2*	5	SYN9183–SYN32947	164.5–177.6	213.1–215.5	3.40	−0.38	5.3
*qSTA9-1*	9	PZE109078278–PZE109082140	72.8–76.4	126.2–130.8	3.26	−0.36	4.7
*qSTA10-1*	10	SYN23550–SYN22965	66.7–73.7	127.6–132.5	5.46	−0.50	9.1

^a^The physical positions of the identified QTLs are based on the B73 reference sequence Version 5.60 (www.maizesequence.org)

^b^A positive value indicates that the allele from CI7 increased the starch content, and a negative value indicates that the allele from K22 increased the starch content

^c^Percentage of phenotypic variation explained by the additive effect of the identified QTL

To further confirm the six QTLs for starch content identified using the BLUP values, we also mapped the QTLs for starch content in CI7/K22 RILs that were grown in different environments (Fig. 3). The association with starch content was stable for all of the QTLs from the RIL populations grown in all three environments. Although the LOD values of some QTLs were lower than the threshold, these QTLs still showed obvious LOD peaks in the RIL when grown in different environments (Fig. 3). In addition to the original six QTLs, one QTL on chromosome 10 was significantly associated with starch content in Hainan in 2013 and had a clear, but weak, LOD peak using the BLUP value (Fig. 3).

In addition to individual QTLs for starch content in maize kernels, the additive × additive epistatic interactions for the identified QTLs in the CI7/K22 RIL population were also investigated. No epistatic interactions were observed (data not shown), indicating that the genetic component of starch content in the CI7/K22 RIL population is mainly characterized by additive gene actions.

#### Identification of candidate genes for starch QTLs

**Fig. 4:**
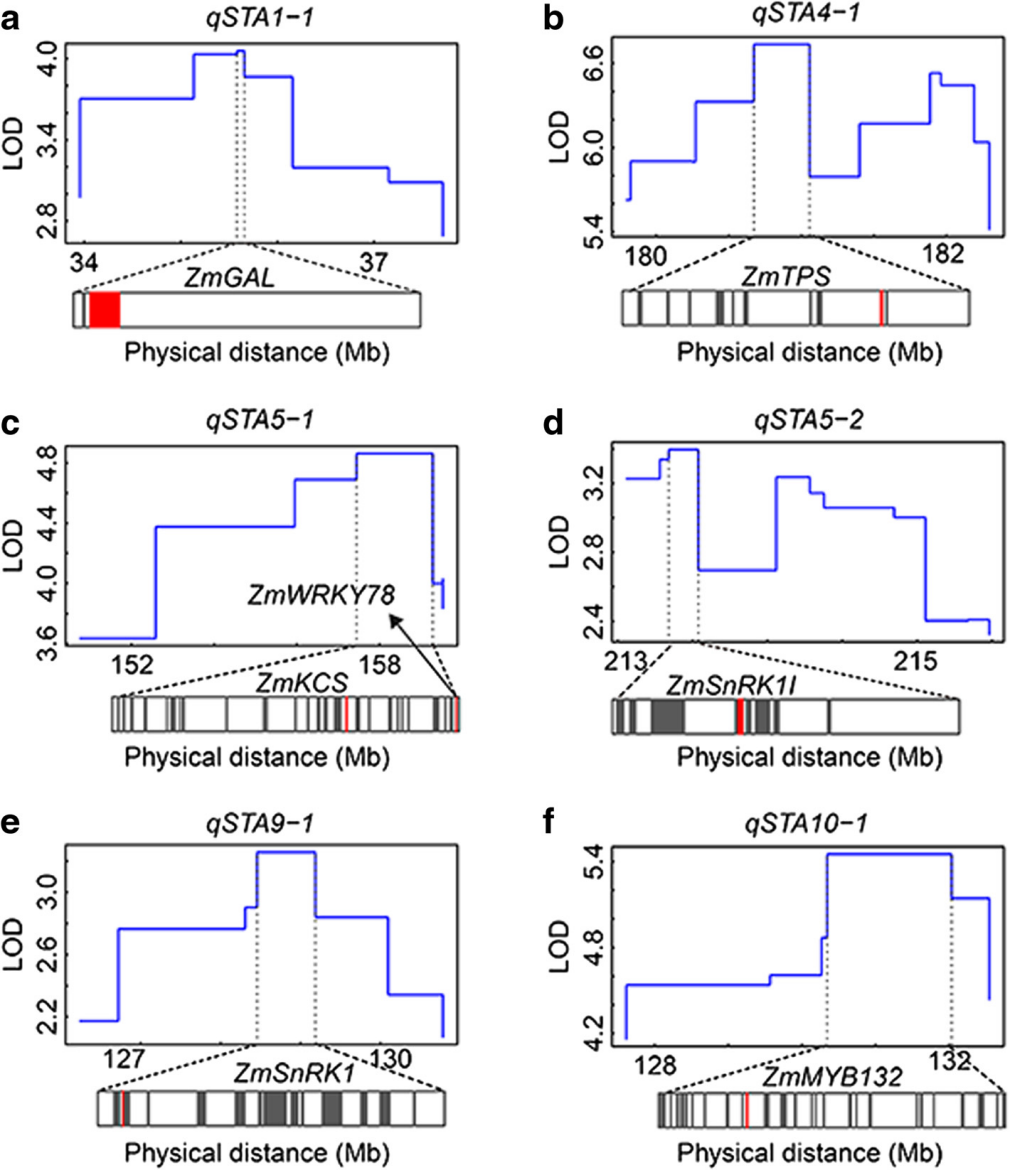
LOD values for QTL bins and representations of genes spanning the peak bin. The blue lines represent the LOD profiles of the bins within a QTL interval. The genes predicted to have putative functions associated with starch content are indicated by red bands; other genes in each peak bin are indicated by gray bands. **a-f** shows the results of *qSTA1-1*, *qSTA4-1*, *qSTA5-1*, *qSTA5-2*, *qSTA9-1* and *qSTA10-1*,respectively.

Combined with the bin map, the intervals containing the six identified QTLs for starch content were narrowed to single bins for each QTL peak (Fig. 4; Additional file 1). The physical distances of the top bins ranged from 81.7 kb (*qSTA1-1*) to 2.2 Mb (*qSTA5-1*), with each bin encompassing 2 (*qSTA1-1*) to 45 (*qSTA10-1*) genes, based on the annotated genes in the B73 reference genome Version 5b.60 (http://ensembl.gramene.org/Zea_mays/Info/Index). The functional annotations of all 144 genes indicated that seven genes, *ZmGAL* (GRMZM2G127123), *ZmTPS* (GRMZM2G151044), *ZmKCS* (GRMZM2G569948), *ZmWRKY78* (GRMZM2G073272), *ZmSnRK1I* (GRMZM2G119769), *ZmSnRK1* (GRMZM2G157743) and *ZmMYB132* (AC206901.3_FG005), were most likely to be the candidate genes for the six QTLs (Fig. 4).

## Discussion

### The genetic component of starch content in maize kernels

**Fig. 5:**
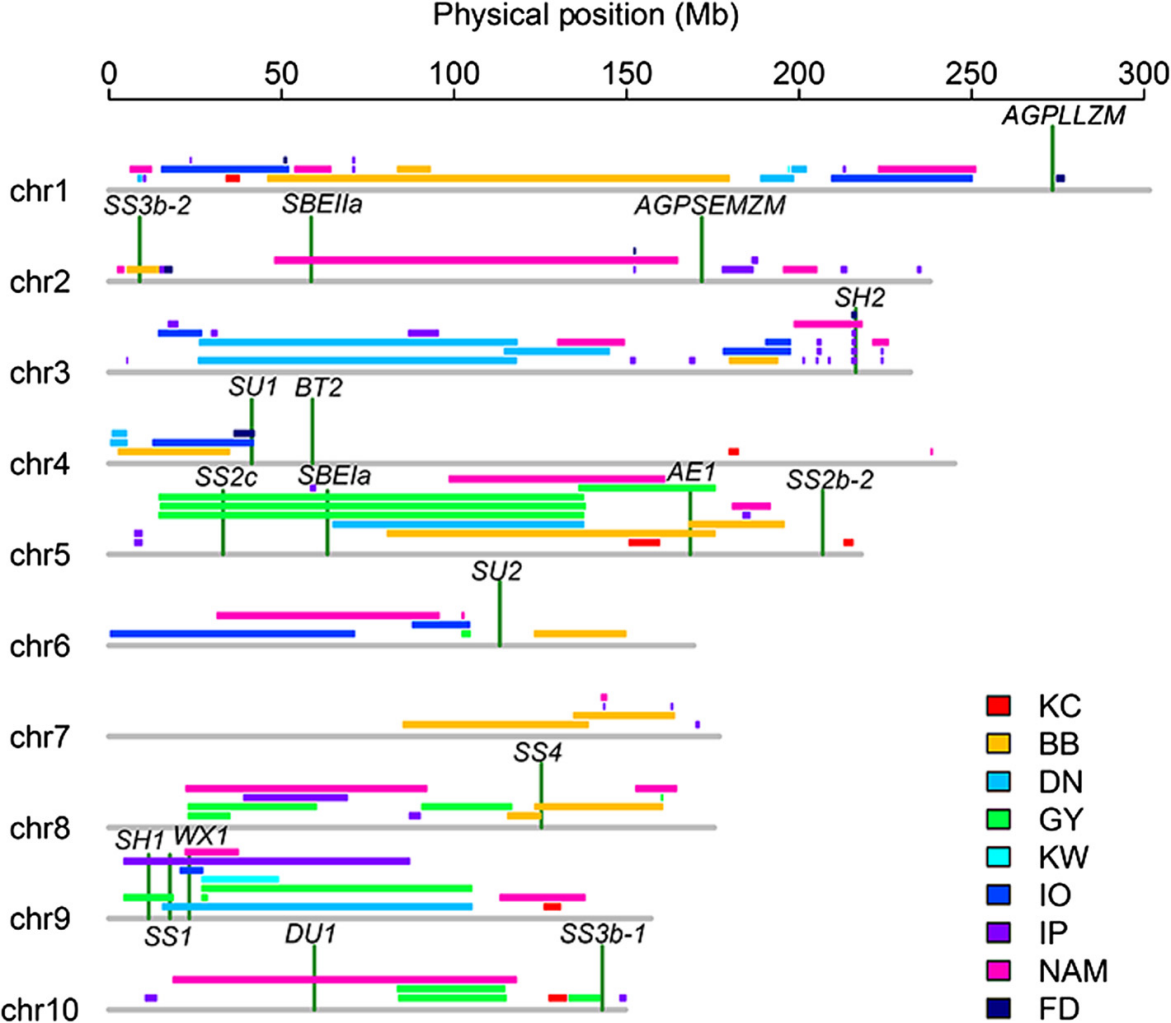
Co-localization of starch content QTLs in maize kernels identified in the current and previous studies. QTLs detected in previous studies are as follows: KC, CI7/K22; BB, By804/B73 [25, 30]; DN, N04/Dan232 [32]; GY, Gy220/8984 or 8622 [27]; KW, W64Ao2/K0326YQPM [31]; IO, Illinois Low Oil/Illinois High Oil [24]; IP, Illinois Low Protein/Illinois High Protein [18, 21]; FD, an early flint line/a late dent line linkage populations [19, 20] and NAM, a nested association mapping population [28]. The vertical dark green lines indicate the positions of 18 well-known genes encoding key enzymes in maize starch metabolism

In the CI7/K22 RIL population, QTL mapping revealed that the variation in the starch content of maize kernels is controlled by at least six QTLs detected by the BLUP value, each accounting for 4.7–10.6 % of the phenotypic variation. All the six QTLs were stable across environments, consistent with high heritability of starch content in this population. However, two QTLs were additionally detected in individual environment (Fig. 3), which can be explained by the interaction between QTLs and environments. We then compared these six stable QTLs with previously identified QTLs based on the available physical locations of the markers and found that 50 % of the QTLs identified in the current study were also detected in previous studies [24, 27–30] (Fig. 5). The QTL *qSTA5-1*, which had the third largest effect, was located in a QTL hot spot, which was reported in multiple studies [27–30]. Interestingly, the top two large-effect QTLs, *qSTA4-1* and *qSTA10-1*, were newly identified in this study. Taking all of the QTL studies together, over 50 loci have been detected for starch content, with one to five QTLs in common genomic regions (Fig. 5), although some loci lacked physical positions for their flanking markers [18–24, 29]. In each population, the number of QTLs for starch content ranged from 3 [31] to 42 [22], and some of the identified QTLs had large effects, with explained starch variations of >10 % [20, 24, 27, 29, 30, 32]. This suggests that a few large-effect QTLs, together with a large number of minor-effect QTLs, mainly contribute to the genetic component of starch content in maize kernels in most biparental linkage populations, which reflects the complexity of starch biosynthesis and accumulation in maize kernels.

Epistasis, the interaction between alleles from two or more genetic loci, is generally considered as a biologically plausible feature of the genetic component of quantitative traits, as the quantitative variation in phenotypes partly results from multifactorial genetic perturbations, such as developmental, transcriptional and metabolic networks [47]. In maize, epistasis gives rise to variation in evolutionary, agronomic and quality traits [24, 45, 48–53]. Previous kernel starch QTL studies in biparental populations also reported that epistasis contributes in part to starch variation in maize kernels [27, 29, 32]. However, epistasis was not responsible for kernel starch variation in the current study, which is consistent with other studies, including a study of a nested association mapping population consisting of 25 RIL populations [28]. This phenomenon might be caused by the small effect of epistasis on starch content, genetic design and/or the power of statistical and computational methods [47]. For *qSTA9-1* and *qSTA5-2*, no epistatic interactions were found based on their candidate gene identifications as *ZmSnRK1* and *ZmSnRK1I,* respectively.

### Association of candidate genes with kernel starch QTLs

Starch metabolism and starch granule size, number and morphology are key factors that influence the starch content in maize kernels. A limited number of genes involved in starch metabolism have major effects on starch quantity and/or quality based on the analysis of well-known maize mutants [2, 3]. The natural association of starch quantity and quality has also been investigated for four genes involved in starch metabolism. *bt2* is significantly associated with starch quantity, *ae2* is responsible for starch quality and *sh1* and *sh2* affect both starch quantity and quality [54]. To further address the natural variation that can be attributed to the known genes in starch metabolism and the possible molecular mechanisms underlying the detected starch QTLs, we compared their physical positions based on the B73 reference genome Version 5b.60 (Fig. 5). Thirteen of 18 genes co-localized with previously identified QTLs, suggesting that these genes might control the co-localized starch QTLs. Unexpectedly, no known genes co-localized with the six QTLs in the current study, indicating that novel molecular mechanisms might underlie these QTLs.

The QTL mapping resolution often depends on the recombination frequency of a population, which is mainly determined by population size and marker density [34]. In a given population, increasing the marker density can reveal the necessary recombination events, increasing the resolution of the genetic map and enhancing the resolution and precision of QTL mapping. An example is QTL mapping using the high-density SNP bin map [42, 55]. The quality and accuracy of the bin map for QTL detection has been validated by studies on multiple traits in rice and maize [35, 40–46, 56]. Thus, we reduced the size of the QTL interval from the original 4.5 to 0.9 Mb in average using the SNP bin map (Additional file 1). The single bins at each QTL peak were considered the fine intervals, as bins with established positions under the QTL peaks exhibited more associations than did those outside of each peak. Furthermore, the accuracy of the associated bins was confirmed by the stability of the QTL peak positions across RILs grown in multiple environments (Fig. 3). The relatively small distance thus allowed us to identify candidate genes for the observed starch content QTLs based on the hypothesis that all of the genes are present in the B73 reference genome. There were 144 genes inside six bins, among which there were seven leading candidate genes for the six starch QTLs (Fig. 4; Additional file 1). However, their association with kernel starch content requires more evidence from such strategies as the further fine mapping of those identified by backcrosses or knock-out or over-expression of the candidate genes.

For *qSTA1-1*, the interval size was reduced to 87.1 kb, which contains only one expressed gene with a functional annotation, *ZmGAL* (Additional file 1). *ZmGAL* encodes a beta-galactosidase, a member of an enzyme group that can release glucose from lactose, a disaccharide that occurs in milk [57, 58]. There are no reports of lactose occurring in plants, meaning that this specific enzyme most likely acts by liberating glucose from another, as yet unidentified, sugar. Glucose has a fundamental role in starch metabolism, and thus variation in *ZmGAL* expression may regulate the amount of glucose, with consequences for starch metabolism. A similar molecular mechanism, based on the function of the leading candidate gene, was investigated for the QTL *qSTA4-1*, which has the largest effect. Of the 12 genes identified within an ~0.5 Mb genomic region, *ZmTPS* was the leading candidate gene for *qSTA4-1. ZmTPS* encodes trehalose-6-phosphate (T6P) synthase in the trehalose metabolic pathway, which shares some common intermediate products, such as glucose, with starch metabolism [59]. Thus, a variation in *ZmTPS* expression will influence starch metabolism. In addition, T6P, a reactant in the reverse reaction catalyzed by T6P synthase, is a sugar signal that is indispensable for carbohydrate utilization and starch pathway regulation in *Arabidopsis* [60, 61]. Leaves of transgenic plants with enhanced T6P have elevated starch levels via a post-translational increase in the redox activation of AGPase, revealing a positive correlation between T6P level, redox AGPase activation and starch content in *Arabidopsis* [61, 62]. Thus, *ZmTPS* was a strong candidate gene for *qSTA4-1*.

The bin at the QTL peak of *qSTA5-1* was the largest among those of the six identified QTLs and harbored 44 genes in the spanning genomic region (Additional file 1). The two genes that are most likely responsible for *qSTA5-1* are *ZmKCS* and *ZmWRKY78. ZmKCS* encodes a member of the 3-ketoacyl-CoA synthase family that catalyzes the condensation of malonyl-CoA with long-chain acyl-CoA, the first committed step in the fatty acid elongation system [63]. Regulating the enzyme activity of 3-ketoacyl-CoA synthase would lead to changes in the amounts of fatty acids with carbon chain lengths of <18, which are the major fatty acids in maize kernel oil. Thus, *qSTA5-1* might have indirect effects on starch content by regulating the oil content that results from variation in *ZmKCS*. In addition, we also considered *ZmWRKY78*, which encodes a member of the WRKY transcription factor family, as a candidate gene for *qSTA5-1*, as transcription factors are key regulators of complex molecular pathways, including the metabolic pathway of kernel composition biosynthesis. The over-expression of *ZmWRI1*, a transcription factor of the APETALA2/ethylene-responsive element-binding protein family, resulted in increased oil content by regulating most steps of oil biosynthesis in maize kernels [64, 65]. Similarly, another transcription factor of the MYB family, *ZmMYB132*, was considered as another leading candidate gene for the second largest QTL, *qSTA5-1*.

In addition to the two transcription factors, two other regulators, a kinase gene, *ZmSnRK1*, and its related interactor, *ZmSnRK1I*, were predicted to be responsible for *qSTA9-1* and *qSTA5-2*, respectively. These predictions were based on the hypothesis that the kinase is essential for signal transduction and regulation and might regulate the metabolic pathway of kernel composition biosynthesis. The size of the *qSTA9-1* interval was reduced from 4.6 Mb to 0.9 Mb and contains 28 genes (Additional file 1). Among these genes, only *ZmSnRK1* seems to be associated with starch content based on the current knowledge. *ZmSnRK1* encodes a serine/threonine protein kinase that plays a key role in the global control of plant carbon metabolism [66]. The over-expression of *SnRK1* in potato tubers causes a significant increase in starch content, resulting from a dramatic increase in the level of expression and activity of sucrose synthase and ADPGase, two key enzymes involved in the starch biosynthetic pathway [67]. For *qSTA5-2*, there were 13 genes in the refined ~0.3 Mb genomic region (Additional file 1). Among these genes, *ZmSnRK1I*, annotated as an interactor of snf1-related kinases, is most likely the candidate gene for *qSTA5-2*. This suggests that *ZmSnRK1I* might indirectly regulate starch metabolism. However, the regulatory mechanisms involved in the transcription of key enzymes in metabolism by *ZmSnRK1* and its interactor in maize remain largely unknown.

In summary, among the seven leading candidate genes for starch QTL in this study, three genes, *ZmGAL*, *ZmTPS* and *ZmKCS*, encoding the key enzymes in non-starch metabolism, might have an indirect effect on starch content by regulating the oil content in maize kernels or have a direct effect on starch content by influencing the amount of the important intermediate product, glucose, in starch metabolism; *ZmWRKY78* and *ZmMYB132*, encoding WRKY and MYB transcription factor family domains, may regulate the expression of key enzymes in starch or the entire metabolism; *ZmSnRK1*, encoding a serine or threonine protein kinase, and its interactor *ZmSnRK1I*, may serve as counterparts that affect the starch content by regulating certain enzyme activities in starch biosynthesis.

### Application of starch QTLs in maize breeding

The starch produced in maize kernels is not only an important carbohydrate source as human and animal diets but also a raw material for industrial and manufacturing applications. To produce starch with properties tailored to food, fuel, fiber or other applications, marker-assisted selection (MAS) is an alternative and efficient strategy for improving starch quantity and quality when QTLs or genes have been identified. Multiple traits have been improved by MAS in maize, such as head smut resistance [68], provitamin A content [69], kernel oil content [70] and haploid induction rate [71]. In the current study, the top two QTLs, *qSTA4-1* on chromosome 4 and *qSTA10-1* on chromosome 10, stable across environments, will be available for the introgression of their favorable alleles to improve the kernel starch content using MAS. Both QTLs explained ~10 % of the starch variation and have additive effects of ~0.5 % in the CI7/K22 RIL population. Whereas, the favorable alleles of these two loci associated with starch content came from different parents. Therefore in order to enhance the starch content into one genotype, it is better to pyramid them in this genotype from different genotypes. Furthermore, the improved resolution of both QTLs, especially for *qSTA4-1*, will increase the reliability of the markers to predict phenotypes using MAS. When the target QTL interval is large, recombination, in some cases, occurs between the marker and gene/QTL because of loose linkage [72–74]. The QTL interval was narrowed to an ~0.5 Mb genomic region, increasing the linkage between the flanking markers and genes/QTLs.

## Conclusions

We identified starch-associated QTLs in a RIL population using a high-density linkage map, refined the identified QTLs based on the bin map and subsequently mined their potential causal genes. The six QTLs accounted for 48.6 % of the starch variation in the CI7/K22 RIL population, with only one QTL explaining >10 % of the phenotypic variation. These findings indicate that large-effect QTLs, as well as minor-effect QTLs, contribute to the phenotypic variation in starch content in the CI7/K22 RIL population. Results from this study improve our understanding of the genetic variants that give rise to variation in kernel starch content, as well as of the possible mechanisms that underlie each QTL, and will provide guidance in manipulating starch quantity and quality by molecular breeding or biotechnology-assisted improvement.

## Methods

### Genetic materials and field experiments

A RIL population consisting of 210 lines was derived from the cross between inbred lines CI7 and K22. CI7 is a high carotenoid and late maturing line introduced from America, which is developed by the USDA-ARS and derived from a backcross of (L317 x 33–16) L317, and K22 is a Chinese elite inbred line derived from a cross between two Chinese inbred lines LK11 and Ye478 [75]. According to phenotypic data of kernel starch content in 474 regular inbred lines [76] in three environments (unpublished data), CI7 has low kernel starch content (around 64 %) and K22 has high kernel starch content (around 69 %). All F_7_ RILs, along with both parents, were grown in a randomized complete block design with one replication in Beijing in 2013, Hainan in 2014 and Neimeng in 2014. Each genotype was grown in a single-row plot having 1 m rows with 0.67 m between rows. In each row, all five ears were self-pollinated and harvested after maturity. Three hundred kernels were bulked for each row, with equal amounts from each harvested ear. Then, 20 representative kernels from each plot were selected from the 300 bulked kernels to measure the starch content.

### Starch content measurement

The starch content in maize kernels was determined using a fermentable carbohydrate assay as described by Zhou and Bao [77]. In brief, the ground powder from 20 kernels was digested with heat-stable α-amylase and glucoamylase. The starch was then fermented into ethanol and carbon dioxide by yeast, and, finally, the starch content was calculated as the weight lost owing to fermentation (CO_2_) and heat (ethanol). All of the samples were measured with two sub-samples analyzed in parallel, and the average was used for subsequent analyses.

### Phenotypic data analysis

All of the statistics were performed using R Version 3.1.1 (www.R-project.org). The linear mixed effect function lmer in the lme4 package of R Version 3.1.1 was fitted to each RIL to obtain the BLUP value for starch content: y_i_ = μ + f_i_ + e_i_ + ε_i_, where y_i_ is the phenotypic value of individual i, μ is the grand mean for all environments, f_i_ is the genetic effect, e_i_ is the effect of different environments and ε_i_ is the random error. The grand mean was fitted as a fixed effect, and genotype and environment were considered as random effects. The aov function in R version 3.1.1 was used to estimate the variances of the starch content. The model for the variance analysis was y = μ + α_g_ + β_e_ + ε, where α_g_ was the effect of the gth line, β_e_ was the effect of the eth environment and ε is the error. All of the effects were considered to be random. These variance components were used to calculate the broad-sense heritability as *h*^*2*^ = σ^*2*^_*g*_*/(*σ^*2*^_*g*_ + σ_e_^2^*/e*) [78], where σ^*2*^_*g*_ is the genetic variance, σ_e_^2^ is the residual error and *e* is the number of environments.

### Genotyping, and the construction of bin and genetic linkage maps

All 210 F_6_ lines in the CI7/K22 RIL population, together with their parents, were genotyped using the Illumina MaizeSNP50 BeadChip, which contains 56,110 SNPs and covers 19,540 genes [79]. The leaf tissue was collected and freeze-dried at −60 °C. Genomic DNA from the leaf tissue was extracted using cell lysis and protein precipitation solution kits (Qiagen, Germany). SNP genotyping was performed on the Illumina Infinium SNP genotyping platform at the DuPont Pioneer Company. PLINK [80] was used to estimate the missing rate, minor allele frequency and heterozygosity for each SNP, and the missing rate and heterozygosity for each line. After quality control, 13,433 SNPs that were polymorphic between the two parental lines were used to construct the genetic linkage map using an economic go-wrong method integrating the Carthagene software [81] in a Linux system with in-house Perl scripts (www.maizego.org/Resources.html). Completely co-segregating markers were assigned to a chromosomal bin, and each bin was considered as one marker.

### QTL mapping

QTL mapping of the starch content was performed using composite interval mapping [82] implemented in Windows QTL Cartographer 2.5 [83]. The scanning interval between markers was set at 0.5 cM, and the window size was set at 10 cM. Model 6 of the Zmapqtl module was selected for detecting QTLs and estimating their effects. A forward-backward stepwise regression with five controlling markers controlled the background from flanking markers. The threshold LOD values to declare the putative QTLs were estimated by permutation tests with a minimum of 1,000 replicates at a significance level of *p* < 0.05 [84]. The confidence interval of the QTL position was determined using the 1.5-LOD support interval method [85]. To further detect the additive × additive interactions between the identified QTLs, multiple - interval mapping in Windows QTL Cartographer 2.5 was performed using the Bayesian Information Criteria as the criteria [86].

### Annotation of candidate genes

Based on the information available in the Gramene BioMart database (ensembl.gramene.org/biomart), the genes within the refined QTL interval and their functional descriptions were extracted. The function of each gene was further confirmed from orthologs in *Arabidopsis* or rice linked in the MaizeGDB database (www.maizeGDB.org). Additional protein prediction information was obtained from the InterPro module in the European Bioinformatics Institute database (www.ebi.ac.uk/interpro/).

### Availability of supporting data

All supporting data can be found within the manuscript and its additional files.

## Additional files

Additional file 1: Table S1. Summary of SNPs polymorphic between two parents in the CI7/K22 RIL population. **Table S2** Summary of the bin and genetic linkage maps in the CI7/K22 RIL population. **Table S3** List of genes within the genomic region spanning the single bin at the QTL peak. (XLSX 21 kb)

## Abbreviations

RIL: Recombinant inbred line
QTL: Quantitative trait locus
AGPase: Adenosine diphosphate glucose pyrophosphorylase
SS: Starch synthase
SBE: Starch branching enzyme
DBE: Debranching enzyme
cM: Centimorgan
SNP: Single nucleotide polymorphism
BLUP: Best Linear Unbiased Prediction
LOD: Logarithm of odds
T6P: Trehalose-6-phosphate
MAS: Marker-assisted selection
